# Neck of Femur Fracture in Young Patients With End-Stage Renal Disease and Hyperparathyroidism: A Report of Three Cases and Proposed Treatment Algorithm

**DOI:** 10.7759/cureus.16155

**Published:** 2021-07-04

**Authors:** Jeffrey J Raj, Ren Yi Kow, Sasidaran Ramalingam, Chooi Leng Low

**Affiliations:** 1 Department of Orthopaedics, Hospital Tengku Ampuan Afzan, Kuantan, MYS; 2 Department of Orthopaedics, Traumatology & Rehabilitation, International Islamic University Malaysia, Kuantan, MYS; 3 Department of Orthopaedics, Hospital Kuala Lumpur, Kuala Lumpur, MYS; 4 Department of Radiology, International Islamic University Malaysia, Kuantan, MYS

**Keywords:** neck of femur fracture, end stage renal disease (esrd), pathological fracture, hyperparathyroid, young adults, bipolar hemiarthroplasty, total hip replacement (thr)

## Abstract

Secondary hyperparathyroidism is a complication arising from untreated end-stage renal disease (ESRD). It can invariably lead to osteoporosis and subsequently cause pathological neck of femur (NOF) fracture. Despite being young, osteosynthesis in neck of femur fractures of these patients often leads to nonunion and implant failure due to severely osteoporotic bone. We present our experience in managing three young patients with ESRD and secondary hyperthyroidism who sustained NOF fractures. All three patients were successfully treated and showed no complication at one year post-operation. Based on our experience and literature review, we propose a simple algorithm to guide the management of these patients.

## Introduction

Hip fracture is a major public health issue in the world [1,2]. It is associated with high morbidity and mortality with up to one-third of patients die within a year after sustaining a hip fracture [2]. While most of the hip fractures occur in the elderly population, 3-10% of hip fractures are attributed to young patients [3-8]. In lieu of their differences in bone physiology and functional status, young patients with neck of femur (NOF) fractures are often treated with internal fixation, either with cancellous screws or sliding hip screws, to preserve the normal hip anatomy and mechanics [3-5]. Garden classification is often used to prognosticate the risk of developing non-union in patients with NOF fractures. They can be divided into four categories, namely: 1) non-displaced, incomplete fracture; 2) non-displaced, complete fracture; 3) partially displaced, complete fracture; and 4) completely displaced, complete fracture [3]. 

End-stage renal disease (ESRD) is a chronic disease of the kidneys with a glomerular filtration rate (GFR) of less than 5ml/min. These patients are often dialysis-dependent with 75-100% of them suffering from metabolic bone disease [9]. The pathophysiology of osteoporosis secondary to hyperparathyroidism is essentially an increase in osteoblastic and osteoclastic activity with abnormal collagen deposition, marrow fibrosis, and high rates of both bone formation and resorption [10,11]. The high bone turnover rate can lead to decreased bone density, predisposing these patients to NOF fractures [9-11]. These patients are often treated with hip replacement surgery due to unacceptable high rate of failure in internal fixation [9,10].

NOF fractures in young patients with underlying ESRD and secondary hyperparathyroidism are uncommon. This specific group of patients, with a contrasting surgical aim, present a therapeutic dilemma to the treating physician. Treating surgeons need to juggle between the aim of preserving femoral heads in these young patients by performing internal fixation for optimal longevity and the aim of preventing unacceptable implant failure by performing replacement arthroplasty. We present our experience in managing three young patients with ESRD and secondary hyperparathyroidism who have suffered from NOF fractures. We also introduce a treatment algorithm upon review of the literature.

## Case presentation

Case 1

**Figure 1:**
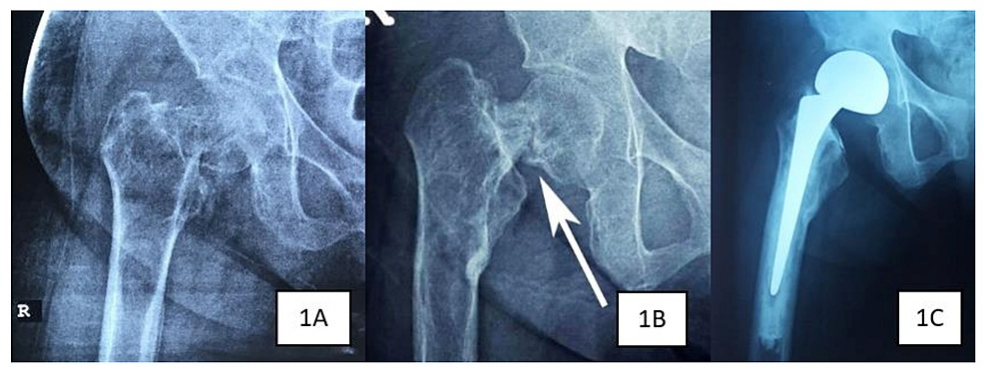
Right hip plain radiographs in Case 1 show intracapsular neck of femur fracture immediately after trauma (1A), post-parathyroidectomy (1B) and after right hip bipolar hemiarthroplasty (1C).

**Figure 2:**
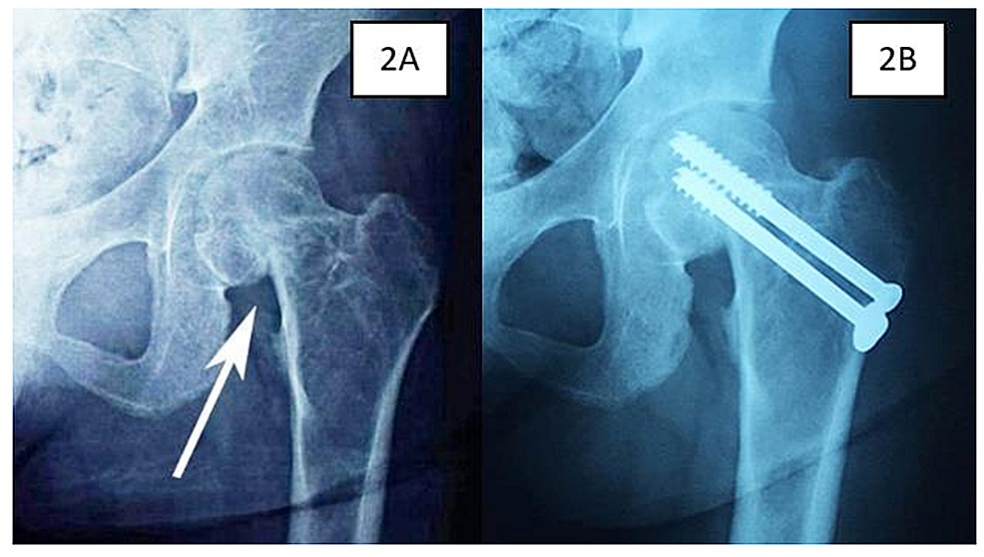
Left hip plain radiographs in Case 1 show intracapsular neck of femur fracture immediately after trauma (2A) and after cancellous screw fixation (2B).

Ms R, a 25-year-old lady with a four-year history of ESRD and secondary hyperparathyroidism, initially presented with right Garden 2 NOF fracture after a trivial injury (Figure 1A). Surgical intervention was postponed due to unavoidable circumstances (Figure 1B). One month later, she presented with contralateral hip pain despite no recent history of trauma or fall. Plain radiographs and computed tomography of the hip both revealed an undisplaced Garden 1 NOF fracture of the left femur (Figure 2A). Her bone mineral density (BMD) had a Z-score of -4.2 which showed severe osteoporosis and intact parathyroid hormone (iPTH) was markedly raised (2593 pg/ml).

The patient was referred to an endocrine surgeon and she underwent a total parathyroidectomy. The iPTH level dropped drastically post parathyroidectomy (<0.3 pg/ml) and her BMD showed significant improvement (Z-score -3.0). Subsequently, a bipolar hemiarthroplasty was done for her right hip (Figure 1C) while the left neck of femur fracture was fixed with cannulated screws (Figure 2B). She was able to ambulate with a walking frame two days post-operatively with full weight-bearing on her right leg. After a period of rehabilitation, she was able to ambulate without support. There was no complication at one year post-operation.

Case 2

**Figure 3:**
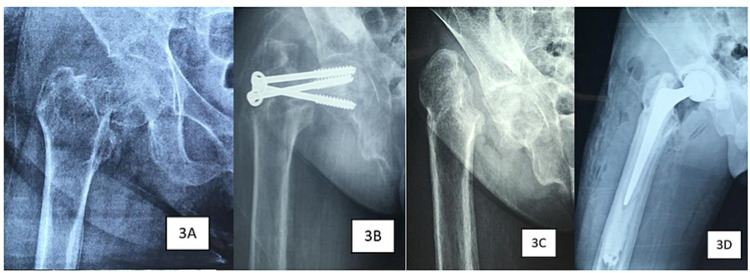
Right hip plain radiographs of the patient in Case 2 show intracapsular neck of femur fracture immediately after trauma (3A), failure of cancellous screw fixation two months post-operation (3B), post-parathyroidectomy (3C) and post-total hip replacement (3D).

Mr K, a 26-year-old gentleman with a six-year history of ESRD and secondary hyperparathyroidism presented with pain at his right hip after a trivial fall. A plain radiograph showed a displaced Garden 2 right intracapsular NOF fracture (Figure 3A). He underwent open reduction and internal fixation in which three half-threaded cancellous screws were inserted. Nevertheless, due to severe osteoporosis, screws loosened and began to cut out after two months (Figure 3B), necessitating removal of the screws. His parathyroid hormone level was 2100 pg/ml and his T-score was -5.2 on the dual-energy x-ray absorptiometry (DEXA), indicating severe osteoporosis. He was referred to an endocrine surgeon and subsequently underwent a total parathyroidectomy.

Six months after the parathyroidectomy, his parathyroid hormone levels dropped to 0.4 pg/ml and his DEXA T-score improved to -2.8. Plain radiographs of the hip demonstrated improvement in his bone density as evidenced by increased trabeculae and cortical thickness of the femur (Figure 3C). He underwent right total hip replacement surgery and he was able to ambulate without complication post-operatively (Figure 3D). There was no complication at one year post-operation.

Case 3

Ms K, a 40-year-old woman with history of hypertension, ESRD, secondary hyperparathyroidism with a baseline serum parathyroid hormone of 199 pg/ml and severe osteoporosis (Z-score: -4.8; T-score: - 5.4), presented to our department complaining of bilateral hip pain following a fall. Previously, she was diagnosed to have right NOF fracture (Garden 3) after a trivial trauma seven months ago which was treated conservatively due to her aversion towards surgical intervention. During this presentation, she complained of bilateral hip pain, causing her to be unable to ambulate.

**Figure 4:**
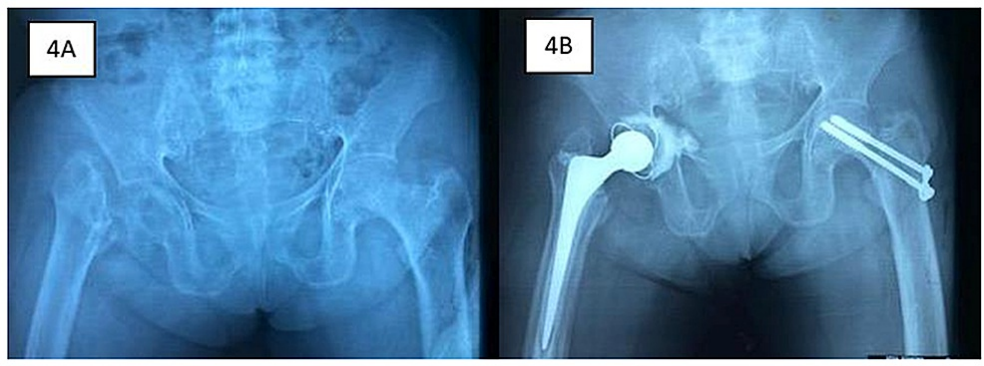
Pelvic plain radiographs of the patient in Case 3 show non-union of right neck of femur (NOF) fracture and non-displaced left intracapsular NOF fracture (4A). She underwent right total hip replacement and left cancellous screw fixation (4B).

The plain radiograph of the pelvis revealed a non-union of the right NOF fracture and an undisplaced Garden 1 left intracapsular NOF fracture (Figure 4A). She underwent right total hip replacement and left cancellous screw fixation (Figure 4B). She was subsequently referred to an endocrine surgeon for parathyroidectomy. Post-operatively, the patient was able to ambulate without aid. There was no complication at one year post-operation.

## Discussion

The management of young patients with NOF fractures differs starkly from the management of elderly patients with similar problems. The main aim of surgical fixation in young patients is to prevent femoral head ischemia by preserving the femoral head vascularity and allow return to pre-injury level of activity [3,9]. This can be achieved by cancellous screw fixation or sliding hip screw, with or without derotational screw [9]. Anatomic reduction and a stable fixation are paramount in treating young patients with NOF fractures to minimize the risk of non-union and osteonecrosis. Aside from implant choice and reduction technique, proximal femur bone density plays a major role in determining the stability of fixation [3]. This is especially true in patients with ESRD where Kalra et al. demonstrated that ESRD patients with NOF fractures who underwent internal fixation had a significantly higher revision rate compared to those undergoing replacement arthroplasty despite the initial fixation was deemed adequate [3]. This is the reason why the patient in case 2 has implant failure after screw fixation of the right hip. 

Treatment of NOF fractures in patients with ESRD and secondary hyperparathyroidism is essentially a race against time. Prolonged immobilization secondary to initial trauma, coupled with an increased bone turnover, often leads to plummeting bone density. These patients often suffer from poor outcomes in terms of higher morbidity rate, prolonged hospital stay, poor functional status and increased mortality [11]. A prospective study by Madsen et al. revealed a significantly higher rate of mortality at one year among patients with secondary hyperparathyroidism [11]. Due to the reduction of bone density, two of our patients presented with contralateral NOF fractures despite no history of trauma in case 1 and trivial injury in case 3.

**Figure 5:**
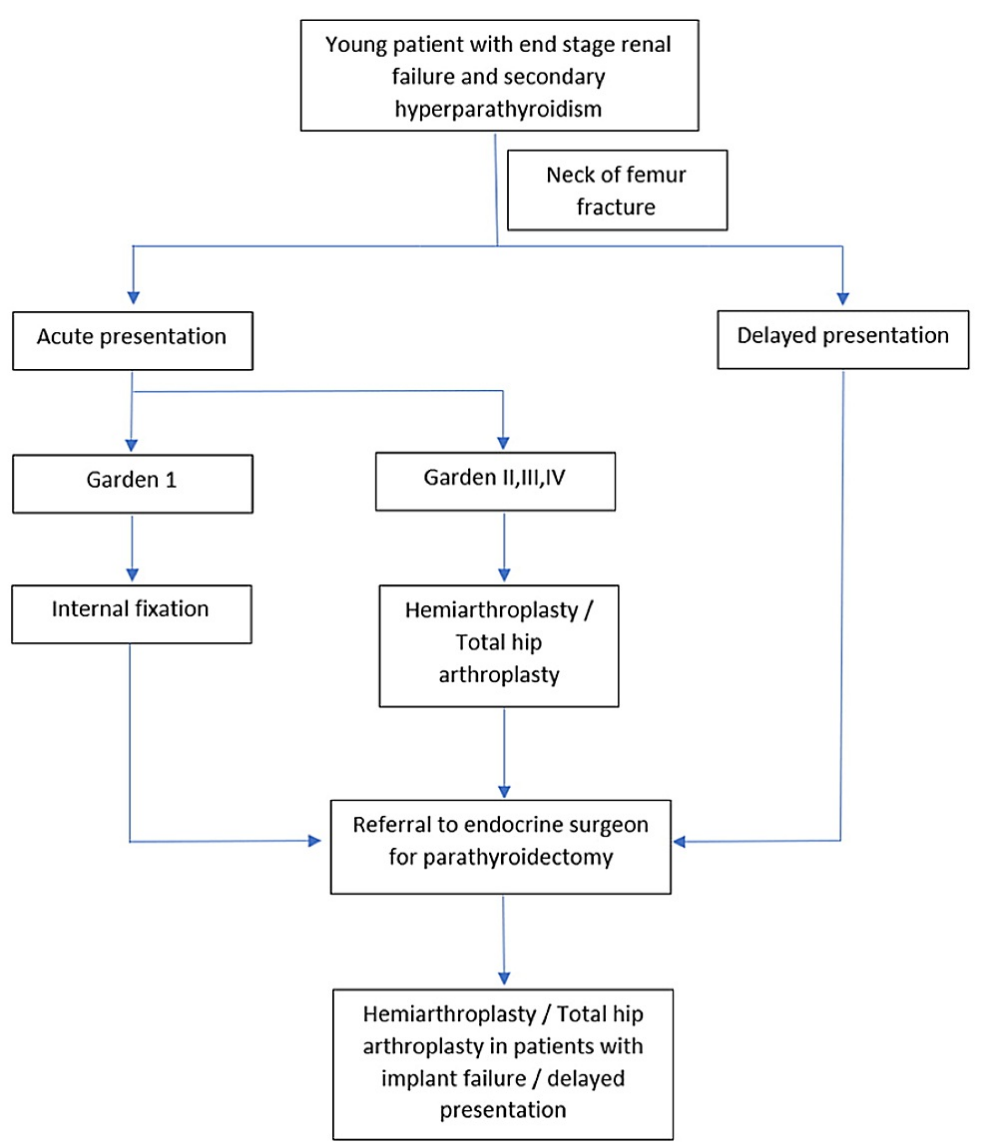
A simple algorithm is proposed to guide the management of neck of femur fracture in young patients with end-stage renal failure and secondary hyperparathyroidism.

Surgical intervention for intracapsular NOF fractures in young patients should be performed as soon as possible in a race to preserve the femoral head. Femoral head vasculature is often compromised in intracapsular NOF fractures. It can be caused by direct disruption of intra-capsular arteries or indirectly secondary to increased intracapsular pressure due to hematoma formation, traction and surgical procedures. Urgent reduction of fracture with minimal manipulation is essential to preserve and restore native blood flow to the femoral head, thus preventing the occurrence of femoral head osteonecrosis [9]. Nevertheless, this treatment plan is not feasible in ESRD patients with secondary hyperparathyroidism. Osteosynthesis will not be stable due to poor existing bone quality of the proximal femur. Femoral head vasculature is further destroyed by intra-operative manipulation for fracture reduction in cases of Garden 2-4 NOF fractures. Hence, in our second patient, despite adequate initial fixation, he suffered from non-union of the NOF fracture. In our opinion, femoral heads in displaced NOF fractures (Garden 2-4) could not be salvaged in ESRD patients with secondary hyperparathyroidism. This group of patients will be benefitted from replacement arthroplasty in the form of hemiarthroplasty or total hip arthroplasty as they will be able to return to normal ambulation and function earlier with less revision surgery [9]. We advocate using cemented replacement arthroplasty in this group of patients for immediate stability. There is a documented risk of loosening of the prosthesis in patients with secondary hyperparathyroidism due to ESRD and hence the need to treat it as an osteoporotic fracture in the elderly with the need for cementing as a precaution [12]. These patients who undergo replacement arthroplasty will be able to weight-bear immediately post-operatively. In contrast to patients who are on non-weight bearing order post-operatively, patients who mobilize early demonstrate an additional functional benefit [13,14]. Besides that, early weight-bearing after replacement arthroplasty has an additional benefit in increasing bone density [14]. In ESRD patients with reduced life expectancy, the quality of life after replacement arthroplasty will be of utmost priority when they unfortunately suffer from NOF fractures [15]. Nevertheless, we still prefer internal fixation for non-displaced (Garden 1) NOF fracture in an attempt to salvage the femoral head as blood supply to the femoral head is relatively preserved. Thus far, there is no literature that compares the outcomes of internal fixation and replacement arthroplasty in management of non-displaced (Garden 1) NOF, hence future studies are needed to answer this question. The treatment outline is summarized in Figure 5.

Irrespective of the initial fracture pattern, timing of presentation and choice of fixation, all ESRD patients with secondary hyperparathyroidism must be referred to an endocrine surgeon for parathyroidectomy after suffering from a NOF fracture (Figure 5). Bone biopsies done by Yajima et al. demonstrated that one week after parathyroidectomy, the plummeting serum parathyroid hormone could suppress bone resorption and increase bone formation [16]. This is essential in maintaining post-operative implant stability, whether the patient has undergone an internal fixation or a replacement arthroplasty. By performing a parathyroidectomy, the incidence of periprosthetic fracture and implant failure will be kept to a minimum. Nevertheless, the optimal timing for parathyroidectomy is unclear and much remains to be revealed on this topic. 

## Conclusions

NOF fractures in young patients with ESRD and secondary hyperparathyroidism should preferably be treated with replacement arthroplasty. We propose a treatment algorithm to guide the management of NOF fractures in this specific population of patients. Further studies should be conducted to verify the feasibility of this treatment algorithm.
